# “Caught in the Middle! Wealth Inequality and Conflict over Redistribution”

**DOI:** 10.1007/s11211-021-00384-x

**Published:** 2022-01-29

**Authors:** Hanna Lierse, Davy-Kim Lascombes, Bastian Becker

**Affiliations:** 1 Center for Inequality and Social Policy, University of Bremen, 28359 Bremen, Germany; 2 Department of Political Science and International Relations, University of Geneva, Geneva, Switzerland

**Keywords:** Wealth inequality, Redistributive preferences, Social classes, Mobility

## Abstract

A vast literature documents that wealth inequality has risen throughout advanced democracies, especially the accumulation of wealth among the rich. Yet, instead of increasing wealth redistribution, governments have done the seemingly opposite. Key to understanding why democratic governments do not increase wealth redistribution in times of rising inequalities is to shed light on the public’s preferences. In this paper, we map the public’s redistributive preferences in fourteen countries based on new survey data. We show that traditional socioeconomic cleavages in preferences for wealth redistribution are undermined by diverging mobility expectations. People who expect to climb up the wealth distribution, mostly lower wealth groups, are less supportive of redistribution than people with high stakes of major wealth losses, mainly upper wealth groups. We show that future expectations among the rich and the poor have a highly moderating role for the class conflict over wealth redistribution. Moreover, the middle class, the decisive group in democracies, is highly unresponsive to future prospects. The findings suggest that the middle class does not have much to lose or to win, and therefore, wealth redistribution is of low salience among this group.

## Introduction

Wealth inequality has risen among advanced industrialized societies and ranks high in comparison with income inequality. In OECD countries, the wealth share for the bottom 60 percent accounts for only 10 percent on average, while the top 10 percent hold over 50 percent. In comparison with wealth inequality, income inequality is a lot lower: The income share for the top 10 only accounts for about 25 percent (OECD, 2018, 2020). As such, the distribution of wealth is a lot more unequal and driven by a concentration of assets at the top (Pfeffer & Waitkus, 2021). While high levels of wealth inequality have always been a characteristic of capitalist societies, the recent rise has puzzled academics and the public alike. After all, democratic societies have policy tools at their disposal, which allow them to tap into the wealth of the rich. Particularly wealth taxes such as the net wealth and inheritance tax are tools that enhance wealth redistribution by placing a high financial burden on the rich (Atkinson, 2015; Piketty, 2014; Saez & Zucman, 2019). Despite rising levels of wealth inequality, we, however, observe the seemingly opposite: rich democracies have cut high marginal tax rates on wealth, which should theoretically be in the financial interest of the majority of society (Hope and Limberg, 2021; Lierse, 2021). One of the key mechanisms for understanding why governments have decreased policies for redistributing wealth is to shed light on the public’s preferences.

In this paper, we explore the public’s preferences for wealth redistribution. Although there is a large literature on the public perceptions of inequality and redistribution, this literature has mainly addressed preferences for income and not for wealth redistribution (Ballard-Rosa et al., 2017; Barnes, 2014; Kelly & Enns, 2010; Lupu & Pontusson, 2011). Wealth, however, differs from income, as it refers to the ownership of assets including financial investments as well as bank savings and housing. Wealth serves as an important financial safety net and at the same time it is a significant marker of social status. While income and wealth can correlate, research also shows that inequality in income and wealth do not necessarily correspond and that wealth inequality is a lot higher than income inequality (Pfeffer & Waitkus, 2021). Hence, the formation of preferences for wealth redistribution takes place in a distinct macroeconomic context.

Based on new survey data from the Unequal Democracies and Inequality in the Minds projects (Pontusson et al., 2020), we explore how attitudes toward wealth redistribution differ among lower, middle and upper classes.^1^ The survey covers thirteen West European countries and the USA for the year 2019. Due to the high concentration of wealth at the top, one might expect the classes at the bottom *and* the middle to be highly supportive of wealth redistribution (Piketty, 2014). And in fact, our analysis shows that people’s wealth position significantly shapes their preferences: Lower wealth groups are more supportive of wealth redistribution than the upper wealth group. However, the evidence also suggests important differences across the three wealth classes. In the lower and the upper classes, future prospects also alter preferences for wealth redistribution, while in the middle-class mobility expectations do not have an effect. It indicates that the large middle, the decisive group in the struggle over wealth redistribution, perceives having lower stakes in the future gains and losses that come along with wealth redistribution. Moreover, we find that the predominantly positive future expectations among the lower-class and negative ones among the upper class blur the traditional class cleavage in the fight over wealth redistribution.

**Fig. 1:**
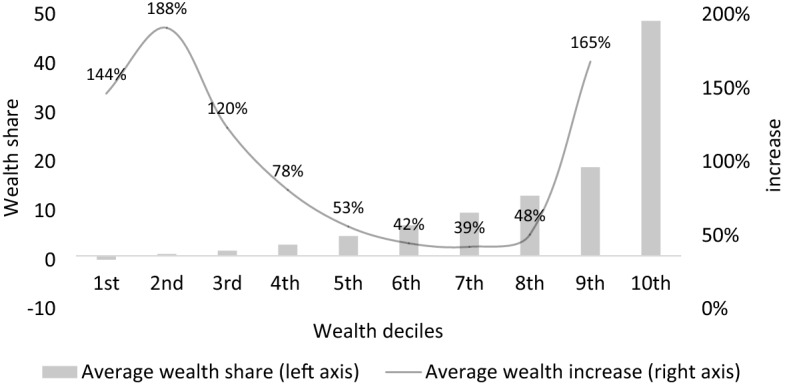
Wealth shares and increases by decile, averages for countries of our survey sample. Source: (OECD, 2018) and own collection

Figure 1 provides some indicative support for the silence of the middle class. The graph shows average real wealth shares for each wealth decile as well as the real percentage changes that come along with moving from one decile to the next. It does not only reveal the limited concentration of wealth in the middle, but it also suggests comparatively low objective wealth changes that come along with moving up or down the wealth distribution in the middle. Being part of the 5th or 6th decile does not substantially change the financial security nor status within society as both groups are able to afford housing, holiday travels and transportation. While the gains and losses are modest in the middle, they are large at the bottom and the top. For instance, moving from the 1st to the 2nd or from the 8th to the 9th decile implies an increase in wealth of over 150 percent. It suggests that for the middle class an upward or downward change has comparatively lower financial implications than for the other two wealth groups. While Fig. 1 shows real implications of mobility, we do not contend that individuals are fully knowledgeable about their objective wealth position nor about the possibilities to achieve future wealth gains. Building on the findings from the literature on income inequality (Gimpelson & Treisman, 2018), we argue that it is the *perceived* mobility expectations that matter.

Before describing our argument in more detail and proposing a set of research hypotheses, we provide a literature review. In section three, we explain our data and methodology including a discussion of how our wealth classes relate to other demographic and socioeconomic variables. This is followed by a discussion of our main empirical findings in sections four, which provides both descriptive illustrations on the public’s support for wealth redistribution and the empirical results from the regression analysis. We conclude in section five by considering the implications of this finding for why the rise in wealth inequality.

## Literature Review and Contribution

Most research on perceptions of economic inequality and redistribution focuses on the concept of redistribution in a broad manner. However, scholars increasingly acknowledge that support for redistribution depends on the specific policy and kind of inequality. For instance, welfare cutbacks are likely to trigger social discontent, while tax cuts can more easily pass without negative sentiments. After all, even if the tax cut is only beneficial to a small rich minority, there are no immediate negative consequences for the rest of society. Similarly, social cutbacks can pass without discontent, if people are largely unaffected. Hence, research has moved toward investigating societal preferences toward specific kinds of redistributive policies (Ballard-Rosa et al., 2017; Edlund, 1999; Limberg, 2019; Roosma & Oorschot, 2020) and taking into account the specific structures of inequalities (Beramendi et al., 2015; Kevins et al., 2018; Lupu & Pontusson, 2011; Pinggera, 2020). While wealth inequality is increasingly recognized as a critical aspect affecting advanced democratic countries, data scarcity has been a limitation to research on this topic. A few scholars have looked into perceptions on the inheritance and the net wealth tax (Atria, 2012; Beckert, 2008; Gross et al., 2017; Korom et al., 2016; Sachweh & Eicher, 2018). However, they only cover a single policy, but do not address more generalizable patterns of preferences for wealth redistribution.

To fill this gap, we explore public preferences toward wealth redistribution by building on the broader literature on preferences for (income) redistribution (Barnes, 2014; Kevins et al., 2018; Lupu & Pontusson, 2011). Wealth, just like income, provides financial security and serves as an important marker of social status. Yet, the different context of wealth inequality, particular the high accumulation of assets at the top, also makes it a particular field of study. Accordingly, the conflict over wealth redistribution should be characterized by a high support for redistribution among those belonging to the lower and the middle classes and high opposition among the top class. Although our analysis confirms that perception of being rich are negatively associated with support for wealth redistribution, we also show that mobility prospects undermine the expected class conflict over wealth redistribution. The preferences of the bottom and the top group are moderated by upward and downward mobility expectations, respectively. Moreover, this centripetal force is reinforced by preference dynamics in the middle. Though most members of the middle class expect to either move up or down in the wealth distribution, these expectations have no influence on their preferences. In the following, we elaborate our argument and formulate research hypotheses with a particular focus on the middle class, the largest and most decisive group in democratic societies (Dallinger, 2013; OECD, 2019).

Research shows that *economic concerns* are an important driver of preferences for redistribution (Bartels, 2005; Page et al., 2013). The main assumption is that people are self-interested and care about their financial gains and losses associated with redistribution. Yet, the way economic concerns are conceptualized differs significantly. The most common way economic interests are measured is by taking their *objective current* income position into account. People at the objective lower end of the income distribution are assumed to be net beneficiaries and hence to be supportive of redistributive policies. In contrast, people at the objective upper end are likely to pay a larger share of the financial burden, and hence, they are more opposed to redistribution. As such, support for redistribution is regarded and also empirically found to decrease with objectively higher level of the income position (Ballard-Rosa et al., 2017; Fong, 2001; Limberg, 2019).

Directly transferring this argument to the wealth context, this implies that preferences for wealth redistribution increase with lower levels of people’s current objective wealth positions. Simply stated: those who hold no wealth are fully supportive of wealth redistribution, those with some wealth are somewhat favorable and those at the very top are opposed to such measures. However, considering that the distribution of wealth differs from income, particularly due to the high accumulation of wealth at the top, scholars have suggested that the majority of society, with the exception of the top, is highly supportive of wealth redistribution (Piketty, 2014). After all, the middle wealth group should have a high interest in aligning their preferences with the lower class to tap into the wealth of the rich.

While there is ample evidence that the current objective economic position is important, several studies have also pointed out high similarities between the preferences of the objectively rich and the poor (Bartels, 2005; Gilens, 2000; Page & Jacobs, 2009; Soroka & Wlezien, 2008; Cansunar, 2021). For instance, Gilens (2000) finds no evidence that such individual economic self-interest plays a role in shaping Americans’ redistributive preferences, and Bartels (2005) shows that many supported Bush’s regressive tax cut although it was not in their objective economic self-interest. There are two main reasons for why the objective current economic position may not be the main point of reference^2^:

First, scholars suggest that it is not the objective economic position, but rather the perceived positions that matters (Cansunar, 2021; Becker, 2021a). Information processing and perceptions are essential ingredients to any model of decision-making. Perceptions and objective conditions are also empirically distinct. Few people know their exact ranking compared to the rest of society. In fact, they do not know about the real extent of inequality and often underestimate overall levels of inequality (Norton & Ariely, 2011; Page & Goldstein, 2016). This is because we tend to compare ourselves to our immediate environment, where households and classes tend to be similar (Hauser & Norton, 2017). While research has pointed to important systematic biases in perceptions, much of the variation in perceptions cannot be explained by objective factors alone.

As perceptions are conceptually distinct and vary independently of objective factors, it is important for empirical studies to consider them separately. Many studies suggest that it is the *perception* of one’s current position—rather than the objective economic position–that primarily influences preferences for redistribution (Evans & Kelly, 2004; Gimpelson & Treisman, 2018). Bobzien (2020) shows that perceptions exert a strong influence even if a wide range of alternative determinants, including political ideology, is taken into account.

Although most studies on perceptions focus on income, similar considerations apply to wealth. While people can gauge the wealth of others to determine their own position, they must do so on incomplete information. Whereas income can mainly be inferred from people’s consumption, wealth is perceived through their physical possessions. This leaves a substantial part of wealth, in particular financial assets, unobserved. Furthermore, people mingle among others with similar levels of wealth, for example because of residential segregation. As such, we believe that many lessons of the income-centered literature apply. While wealth perceptions are likely to be biased systematically, they can only partly be explained by objective differences. Following the recent literature, we therefore focus on people’s perceptions and not on their real position.

Second, while the current perceived wealth position is certainly important^3^, the future mobility expectations play an important role in determining redistributive preferences. The existing literature has highlighted that individuals do not only value their current position but significantly rely on their evaluations about the future (Becker, 2021b; Benabou & Ok, 2001; Cojocaru, 2014; Piketty, 1995). Although people currently rank low in the economic distribution, it is possible that they feel confident about the future and assume to receive a considerable rise in incomes and accumulate wealth in the long run. People with positive expectation about their future mobility are likely to differ from those with no or negative expectations. Such expectations can critically alter the relationship of the current economic position on support for redistribution, particularly because research shows that people tend to over-estimate social mobility expectations. That is, the majority of society in fact believes that they will be better off in the future (Bjoernskov et al., 2013; Engelhardt & Wagener, 2014).

This finding is also important in the context of wealth redistribution. Wealth gets generated through the accumulation of incomes, capital gains or inherited wealth, which tends to increase in the future. However, not everyone has the same positive expectations and some might even fear substantial losses. We expect such differences to be important in shaping the public’s preferences for wealth redistribution. Imagine a person who believes to be poor right now because all the capital has been invested into a startup. The indebtedness is due to a bank loan for a new venture, which is likely to generate prosperous incomes and to contribute to future wealth. This is quite a different situation from feeling poor without having any future prospects. Both feel poor, but their future prospects for the accumulation of wealth are entirely different and this probably influences their preferences for wealth redistribution. It is likely that upwardly mobile individuals are more interested in maintaining wealth differences, while downwardly mobile individuals seek to reduce them.

Such future expectations about the accumulation of wealth are, however, unlikely to be distributed equally among society. First, different effects at the top, middle and the bottom of the relative wealth distribution are likely to alter each group’s mobility expectations. First, we suggest that people who form part of the lower wealth group are more likely than the rest to have upward mobility expectations. Although studies show that poverty is difficult to escape due to self-reinforcing effects, most people tend to over-estimate future mobility possibilities (Bjoernskov et al., 2013; Engelhardt & Wagener, 2014). Particularly, lower wealth groups are likely to perceive a substantial room for upward mobility, because even small wealth increases are associated with considerable gains in social status. Amassing small savings or wealth items, such as a car, television or a small apartment, can result from economic windfalls and increase financial security and boost social status compared to their peer group.. By contrast, the top, who owns a lot of wealth, also has a lot to lose. Although the rich are likely to further accumulate wealth through financial investments, increasing one’s financial wealth does not imply that the rich also perceive to become relatively richer. In contrast to the poor, the rich have little space to move higher on the wealth distribution, but plenty to fall down. Ironically then, the top wealth group is most likely to have the lowest future expectations.

But what about the middle class? Do they share the hopes of the lower or the fears of the higher wealth class? We expect the middle class to have a mix of both upward and downward mobility prospects. After all, it seems likely that the middle is more mixed, i.e. some expect positive and others negative developments of their future wealth position. But yet, these concerns—whether positive or negative—are less consequential in the middle in comparison with the other two groups. They do not share the high hopes of the lower class as they already live comfortable lives and wealth changes have less of an impact on their lifestyle. Although wealth increases might allow them to buy a bigger house or car, this does not entail a substantial change in their social status. Put differently, increases in relative wealth are likely to remain unnoticed. Similarly, they do not share the high risks of the top wealth group as they tend to make more conservative investments and hence have less to risk. They do not fear losing their homes or savings, which they invested as a safety net for dire times or old age. In other words, although the middle class has mobility expectations, the gains and losses do not substantially change their wealth and social status—they are comparatively small. In sum, expectations of upward and downward mobility are mixed in the middle group, while downward expectations define the upper group and upward expectations the lower group.

*Hypothesis 1* Compared to the middle class, the lower wealth group is more likely to have upwards expectations and the top wealth group more downward expectations.

How do mobility expectations alter the public’s preferences for wealth redistribution? It is our main argument that future wealth prospects among the three groups moderate the traditional class conflict over redistributive preferences. While we expect that positive future expectations are generally associated with greater support for wealth redistribution, negative expectations with lower support, the scope of this effect differs among the three groups in a way that they are caught in the middle. In the lower and the upper group, mobility expectations—whether positive or negative—tend to have large implications for their future. In both groups, wealth changes can induce considerable changes in social status. And whereas the low wealth group can hope to acquire a certain degree of financial security even through small wealth increases, the fearful rich might regard wealth redistribution as an important safety net. It is also important to note that even if wealth increases among the low wealth group are often unlikely to be taxed, individuals might still be concerned about the possibility of taxation. All in all, in both groups—the bottom and the top—mobility expectations are likely to change their preferences for redistribution in a way that blurs the traditional class conflict.

In contrast to the low and the top wealth group, future hopes and fears do not alter the preferences for wealth redistribution among the members of the middle class. Even when individuals of the middle believe to be better or worse off in the next five years, such expectations have limited consequences. As shown in Fig. 1, the wealth changes associated with possible gains and losses are relatively small and will not substantially alter their social status. While we do not contend that they know or are entirely unconcerned about the real financial implications, the middle class feels comfortable in their position anticipating to become neither rich nor poor despite their mobility expectations. In other words, although they believe to move up or down the wealth ladder, the mobility expectations do not significantly change their preferences for wealth redistribution.

*Hypothesis 2* Compared to the middle class, the lower and upper wealth groups mobility expectations have a stronger effect on redistributive preferences.

What are the implications for this? In line with Ares (2020), our hypotheses suggest that mobility expectations undermine the expected group conflict over wealth redistribution. Due to mostly positive future expectation, the lower class is less likely to push for wealth redistribution, while high financial risks at the top make the rich more likely to support state intervention in the distribution of wealth. Finally, the middle class is comfortable and largely unresponsive to future changes. This certainly does not imply that they do not support wealth redistribution, but it does suggest that their economic hopes or fears play no role in their formation of policy preferences.

## Data and Methodology

We test our two hypotheses based on new survey data collected in 2019 by the Inequality and Politics Study (IAP) (Pontusson et al., 2020). The dataset addresses key issues of perceptions of income and political inequality as well as preferences for income and wealth redistribution. A minimum of 2001 respondents representative of the general population answered an online questionnaire in fourteen countries: Austria, Belgium, Denmark, France, Germany, Italy, Ireland, the Netherlands, Portugal, Spain, Sweden, Switzerland, UK and the USA. Quotas were implemented by region, gender, age, income and level of education.^4^

From the IAP dataset, three variables are of crucial interest for our study. First, as regards our dependent variable, we use a survey question, which measures individuals’ preferences for wealth redistribution. Respondents were asked to state their position on redistribution of wealth from the rich to the poor on a 10-point Likert scale where 0 means that they are “fully opposed to the redistribution of wealth” and 10 that they are “fully in favor of the redistribution of wealth.” Variations in respondents’ answer are our main focus of interest throughout the analysis with higher numbers suggesting higher support for wealth redistribution.

As regards the main independent variables of interest, we use two survey questions to generate two empirical indicators: The first survey question asks about people’s perceived current position in the wealth distribution, and the second addresses the expected position in five years. For both variables, respondents were asked to position themselves on a scale indicating the share (in percent) of households they estimated poorer and the share they estimated richer than their own. To lighten the cognitive loads of these positioning tasks, the two survey questions used an interactive scale indicating simultaneously the estimated share of poorer and richer household. Moreover, the question on the expected position followed directly the question on the perceived current position (see vignettes in the appendix, Fig. 5). From this, we create the variable perceived current position (in percentile), which ranges from 1 to 100 with lower numbers indicating perceptions of owning relatively less wealth than the rest of society and is included as a control in Model 1 of our analysis.^5^

Based on this variable, we construct the *wealth class* indicator, one of our two main independent variables. We categorize those who perceive to fall in the lowest 30 percent as belonging to the lower class, those ranking between the 31^st^ and the 70^th^ percent as the middle and those in the top 29 percent as the upper wealth class. Of course, the delimitation of our three wealth classes is partly arbitrary and draws boundaries that are blurred in social reality. However, this measurement is largely inspired from other methods (Dallinger, 2013) considering the middle group being the largest in contemporary democracies (OECD, 2019). Because our measure rely on a perceived position and not an objective one, the choice of the thresholds must also take into account respondents’ answer distribution. With our measure, 68 percent of the respondents are in the middle, 23 percent in the lower class and 8 percent in the upper class (see Table 1).^6^ For a further discussion, you can consult Sect. 4.1, which describes the empirical composition of our wealth classes.

**Table 1:** Socioeconomic and occupational composition of the three wealth classes

	Permanent contract	Employed full-time	Manual worker	University degree	Household income	Female	Average age
Lower (23.72%)	66%	27%	32%	21%	d1–d3: 54%	54%	45
					d4–d7: 37%		
					d8–d10: 9%		
Middle (68.18%)	77%	47%	21%	30%	d1–d3: 21%	52%	44
					d4–d7: 46%		
					d8–d10: 33%		
Upper (8.10%)	77%	50%	15%	45%	d1–d3: 18%	40%	45
					d4–d7: 26%		
					d8–d10: 56%		

d1–d10 refers to the respective income decile.Source: IAP 2019

Second, to measure the *prospect for mobility,* we use the percentile difference between the estimated position of the household “five years from now” and the perceived position at the present time. It ranges from -100 to + 100 with negative numbers indicating an expected decline in the wealth position and positive ones a higher position in the future wealth distribution. The two indicators, 1. wealth classes and 2. prospect of mobility, are our key independent variables to test our above-described hypotheses.

Besides the two main independent variables of interest, we control for a number of other individual-level characteristics, which have been found influential in the literature. First, we use the household income decile after tax, which ranges from 1 to 10, with lower values indicating lower income deciles. Second, we use a dichotomous variable for gender, where a 1 corresponds to being female. Third, we add an education variable, which is 0 if no university degree has been obtained and 1 otherwise. Forth, we control for age (in years), fifth, for union membership, where 0 means that one has never been a member of a trade union and 1 otherwise. Sixth, we include a variable for being native born, where 0 means born in the respective country and 1 refers to being born abroad. Finally, for the employment status, we include an indicator, which considered the following categories: full-time employment (reference category), part-time employment, self-employment, unemployment, full-time parent/homemaker, full-time student and retired.

To explore how support for wealth redistribution varies between different groups of society, we start by providing descriptive evidence in the next section. This is followed by a discussion on our empirical results based on linear regression models with country fixed effect. The outcome variable is the above-described survey question on preferences for wealth redistribution. To test our hypotheses (Table 1), M1 focuses on the variable prospect of mobility to take into consideration the role of mobility expectation on preferences for wealth redistribution. It also includes individual’s perceived current position in the wealth distribution to gauge whether respondents with lower/higher wealth positions tend to be more/less supportive of wealth redistribution. The model M2 interacts the three wealth classes with expected mobility. This allows us to explore our second hypothesis, which postulates that the middle group is less responsive to gains and losses associated with future changes, whereas the bottom and the top classes are more likely to react to mobility expectations. For robustness checks, we run M2 with different operationalizations of wealth class: 1. increasing the size of the lower and upper classes and decreasing the middle-class one and 2. increasing the size of the middle class and decreasing the size of the lower and upper class. The findings are in line with the main models included in the text (see appendix, Tables 4 and 5).

## Empirical Discussion: Wealth Class, Mobility and Preferences for Wealth Redistribution

In the first part of our empirical discussion, we shortly describe the empirical composition of our wealth classes. This gives a better idea of how they relate to other demographic and socioeconomic variables, and thus other class concepts. In the second part, we provide descriptive illustrations assessing differences in mobility expectations across the three wealth classes as suggested by hypothesis one. In the third part, we discuss the empirical evidence of our regression analyses testing our second hypothesis.

### Wealth Class Composition

The most widely used class schemes focus on individuals’ employment position in the labor market (Erikson & Goldthorpe, 1992; Oesch, 2006). Class scholars suggest that the occupational structures generate distinct social groups experiencing different economic advantage and levels of protection from the labor market. Consequently, an economic class is broadly defined as a group of individuals, who, due to a common economic position, share understandings, status, norms and interests (Kocka, 1980). Other works more conveniently use the socioeconomic status (Dallinger, 2013; Lupu & Pontusson, 2011) as a proxy to define class. In this section, we briefly highlight the similarities and differences of our wealth class with other commonly used measure such as socioeconomic status (Dallinger, 2013; Lupu & Pontusson, 2011) and occupational position (Abou-Chadi & Wagner, 2019; Ares, 2020; Oesch, 2006; Oesch & Rennwald, 2018).

Table 1 describes and compares the share of respondents from the lower, middle and upper wealth classes with respect to their employment situation, occupation, level of education, income position, gender and age. The table indicates that two-thirds of the respondents fall in the middle wealth class, 23.7 percent into the lower and 8 percent into the upper wealth class. Respondents who perceive to be at the bottom of the wealth distribution also tend to have lower socioeconomic status. First, they tend to have lower incomes as 54 percent are in the three lowest income deciles. Moreover, they are more likely to be manual workers and less likely to have a permanent contract compared to the two other wealth groups. Furthermore, respondents of the middle and upper classes are more likely to have permanent contracts. However, those in the upper wealth class tend to earn more as 56 percent of them are in the three highest income deciles. They are also on average more educated and tend to have more valued occupations than those in the middle wealth class (see appendix, Table 2). About 28 percent of the upper wealth class respondents have professional and technical occupations, and 19 percent have a higher administrator profession, while those numbers fall to 17 and 7 percent for the respondents in the middle class.

Overall, the composition of the three wealth classes indicates that our concept of wealth classes partly overlaps with other conceptions and measurements of economic classes. Respondents in the upper class being more educated, earning more and having better occupations, and at the opposite, lower-class respondents, earning less and being more likely to be manual workers. However, our wealth class measure, relying on perceptions, also allows significant deviations from other socioeconomic and occupational class measurements. A large share of top earners, about 43 percent, falls in the lower and middle wealth classes and about 68 percent of the manual workers are part of the middle and upper wealth classes. Knowing the socioeconomic and occupational composition of the three wealth classes, the following sections focus on wealth classes' role and prospect of mobility on preferences for wealth distribution.

### Descriptive Evidence

Hypothesis 2 suggests that the effect of mobility expectation varies across wealth classes. As such, it complements two baseline models describing the effect of perceived position (Gimpelson & Treisman, 2018) and prospect of upward mobility (Benabou & Ok, 2001) on preferences for redistribution. Before turning to a direct test of H2, this section provides an illustration of the two baseline models and shows descriptive evidence confirming our first hypothesis.

**Fig. 2:**
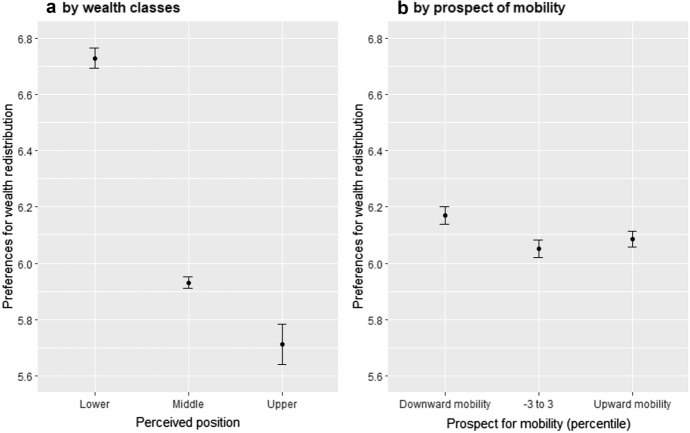
Preferences for wealth redistribution by wealth classes and mobility expectations

First, Fig. 2a shows whether support for redistribution differs by perceived wealth position by plotting the average preferences for the three wealth classes. As expected, preferences for wealth redistribution increase with lower positions in the wealth distribution with the bottom having highest preferences for wealth redistribution (6.7), being followed by the middle (5.9) and the upper class (5.7). Surprisingly, however, the preference gap between the lower and the middle is larger than between the middle and the upper class. The support for wealth redistribution drops on average by 0.8 points on our 1–10 scale moving from the lower to the middle class. By contrast, moving from the middle to the upper class, support for wealth redistribution only declines on average by 0.2 points. This is somewhat surprising. Given the high concentration of wealth at the top, we would have expected the middle class to align their preferences more with the lower class. After all, both groups would benefit from tapping into the wealth of the rich.

To understand why the preferences of the middle class align more with the rich than the poor, we need to revert to other explanations (see literature review). We proposed two possible reasons: First, not only the current wealth position is of importance, but also the expected future gains and losses matter. That is, support for redistribution is likely to increase with negative mobility expectations. Those who fear future losses are more supportive of government intervention, whereas those who expect future gains are more resentful to financially payments. Moreover, we argued that the effect of mobility expectations depends on the wealth class (hypothesis 2). Particularly, we suggested that the prospect of downward mobility is largest among the rich as it can lead to a large decrease of wealth and social status, while prospects of upward mobility are largest among the poor. In turn, the middle class’ (downward or upward) mobility expectations are more mixed and comparatively lower.

Figure 2b provides descriptive evidence for the POUM hypothesis, which posits that mobility expectations influence public support for wealth redistribution. The graph shows the average preference for redistribution for the respondents expecting to move downward in the wealth distribution (29.8% of the respondent), to move upward (38.2%) and those expecting little or no movement in the coming 5 years (less than 3 percentiles, 32.5% of the respondents). In line with the literature, individuals who expect to move downward are on average more supportive of wealth redistribution in comparison with the upward mobile group. Curiously, however, those not expecting to move do not seem more supportive of redistribution than those with more positive future prospects. Moreover, individuals’ mobility expectation seems to have much less effect than their wealth classes on their preferences for redistribution.

**Fig. 3:**
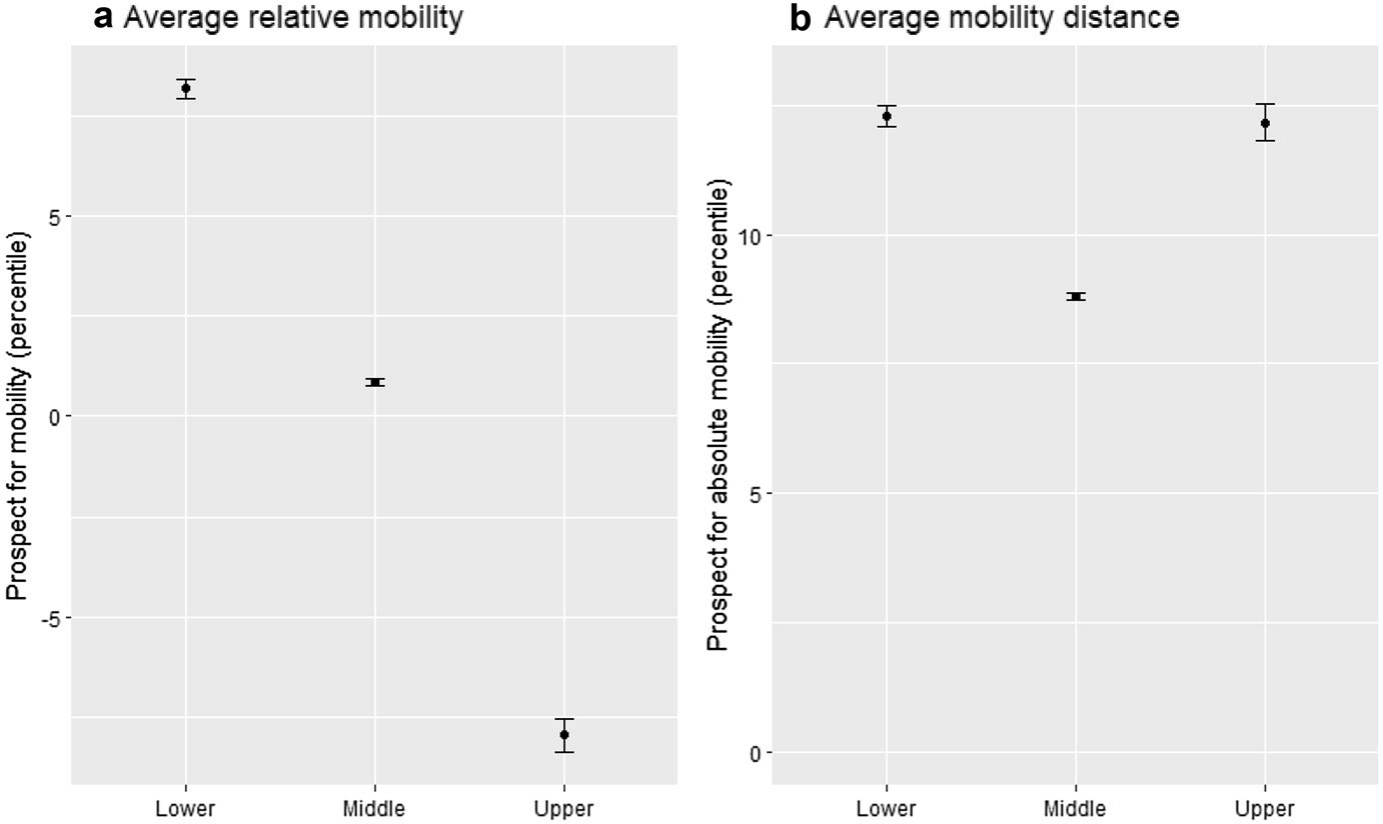
Mobility expectation by wealth classes

To make sense of the observation that people with no mobility expectations are less supportive of wealth redistribution than those with upwards prospects, we need to better understand differences between the three wealth classes. It thus relates to hypothesis 1, which suggest that the economic position shapes upward and downward expectations. We provide descriptive evidence with Fig. 3, which illustrates differences in mobility expectations between the three wealth classes, the lowest, the middle and the top, respectively. The first panel describes the average relative mobility while the second measures the average mobility distance. For example, a respondent expecting to move down by 10 percentiles would have a score of -10 percentiles (measure used in Fig. 3a), which correspond to a 10 percentiles point distance from her position of origin (measure used in Fig. 3b).

In fact, Fig. 3 confirms our proposition of H1 and highlights differences in mobility expectations among the three wealth groups. First, Fig. 3b shows that individuals in the upper and the lower group expect to move on average about 12 percentiles in the coming five years in contrast to the middle who shows a slightly lower average of 9 percentiles on our + 100 to – 100 mobility scale. While this indicates greater mobility outlooks at the top and the bottom, the expected movement by the middle is not negligible. The fact that individuals in all wealth classes expect changes in their wealth position is important to our argument. After all, we proposed that status concerns are more pronounced among the bottom and the top because individuals in these two classes react more strongly to their expectations. In the middle group, we expect no such reaction, not because people do not expect any mobility, but because status concerns are less pronounced, and therefore, mobility expectations do not translate into preference changes.

Second, Fig. 3a shows that the average direction in which individuals of each group expect to move differs in a way to reflect a regression to the mean or a centripetal pattern. Whereas individuals at the bottom class expect to move upwards by on average 8.2 percentiles, those at the top expect to move downwards by about the same amount. In the middle class, negative and positive expectations are similarly present, somewhat canceling each other out in the aggregate, with an average upward mobility of 0.85 percentiles. In other words, our observations suggest that individuals in the lower wealth class are also the most optimistic group concerning their future position. Therefore, an important share of those expecting to move upward (Fig. 2b) coincides with the lower class, which can explain, why we observe a relatively high level of preference for redistribution among this group. By contrast, a large share of those with downward prospects overlaps with individuals from the upper wealth group. As the rich have a lot at stake and are particularly fearful of downward movement, this group is on average more supportive of wealth redistribution than those who expect very little or no mobility. In a way, relatively high preferences for some governmental redistribution serve as a form of insurance: Wealth redistribution can counter fearful concerns of stark losses. By contrast, people who do not expect to move much have very little at stake and hence are less concerned about insuring potential losses. Altogether, the descriptive patterns suggest that the effect of mobility expectations on wealth redistribution varies between classes as proposed by hypothesis 2. In the following section, we provide further insights on this aspect based on the statistical analyses.

### Empirical Evidence

Table 1 summarizes the main findings of our linear country fixed effects. The full model can be found in the appendix (Table 3). Model 1 (M1) shows the results including the current perceived position in the wealth distribution and mobility expectations, whereas Model 3 (M3) tests the interaction effect (hypothesis 2). The findings of the statistical analysis largely support the descriptive evidence discussed above. First, M1 shows that individuals current wealth position has a negative and statistically significant effect on preferences for wealth redistribution. The higher individuals perceive their position in the wealth distribution, the less they favor redistributive measures. For instance, moving from the perceived 10^th^ percentile position to the middle (50th percentile), we expect a decrease in support for wealth redistribution by about 0.5 points of our dependent variable and about another 0.5 points reduction moving to the 90th percentile. This finding is largely in line with existing literature, which shows that economic concerns are a decisive factor driving public support for income redistribution (Duman, 2019).

M1 also tests the POUM hypothesis, which posits that individuals expecting to move up in the wealth distribution are less supportive of redistribution and vice versa. The estimations confirm our theoretical expectation and the descriptive support provided in the previous section, as the effect of mobility prospects is negative and statistically significant. It suggests that the higher the perceived future wealth gains are, the lower is the demand for redistribution. In fact, our findings indicate that for each expected percentile gain, individuals decrease their preferences for redistribution by 0.01 point. This means, for instance, that an individual, who expects to move upwards from the first wealth decile to the fifth, is by 0.42 points less supportive of wealth redistribution than someone who expects to stay in the lowest decile. This finding is interesting insofar as future mobility expectations to some extent undermine the class struggle for wealth redistribution. It implies that poor people are not necessarily supportive of wealth redistribution due to their upward mobility expectations and the rich are not necessarily against it due to fears of future losses of wealth and status.

**Fig. 4:**
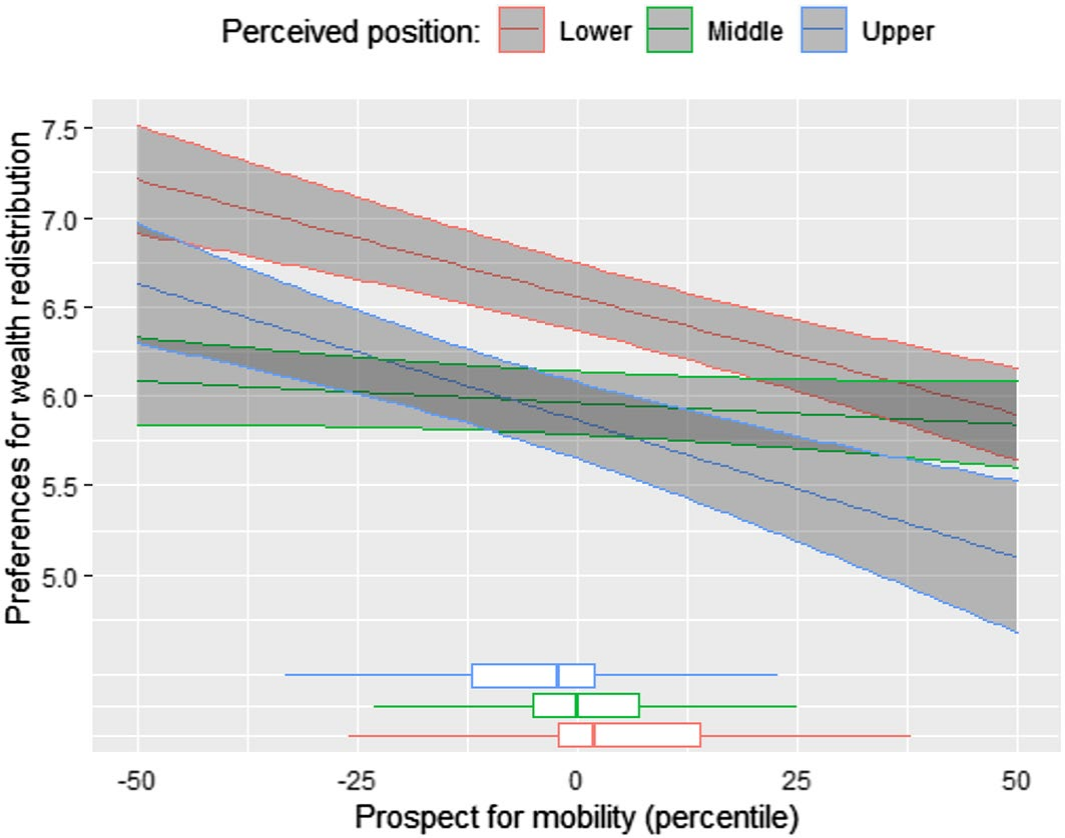
Predicted preferences for wealth redistribution and prospect of mobility by wealth class

Finally, M2 tests our second hypothesis, which suggests that the effect of mobility expectations differs between the three wealth classes. To test this assumption, our second model interacts the prospect of mobility with the three perceived wealth classes—with the middle being our reference group. The results are in line with the expectations and show that both lower and higher wealth classes are significantly more sensitive to the effect of prospect of mobility.^7^ Moreover, the observed effects are particularly large. Mobility expectation for the lower and upper classes impact on preferences for redistribution has about the same magnitude than the objective household income position. In other words, for the upper and lower groups expecting to climb the wealth ladder by one decile has the same effect than being higher in the income distribution by one decile. To better illustrate the impact of the prospect of mobility on the three classes, Fig. 4 plots the predicted preferences (based on Model 2, Table 1) and mobility expectations across wealth classes.

The results of Fig. 4, showing how the impact of the prospect of mobility varies between the three classes, are striking. For the rich and the poor, the prospect of upward mobility has a strong negative effect on preferences for wealth redistribution. That is, preferences for redistribution drops significantly by 0.21 points (6.16–5.95) and by 0.31 points (5.40–5.09) for respondents in the lower and upper class that expect to move upwards by 30 percentiles. However, for the middle class, prospects of mobility have virtually no effect on their preferences for wealth redistribution. The graph indicates that for the middle wealth class a prospect of upward mobility of 30 percentiles decreases their preferences for wealth redistribution by only 0.06 points (5.95–5.89). In other words, the mobility expectations have a strong and significant effect on the rich and the poor, and a negligible one for the middle class. Moreover, the mobility expectation differences across wealth classes amplify the interaction effect, as poor and rich have more mobility expectations. The middle, which should theoretically be not only highly supportive of wealth redistribution, but also highly responsive to changes associated with the distribution of wealth, seems to be rather indifferent about possible future losses and gains. As argued above, one explanation for the insignificant effect of mobility expectations on the middle is that this class is a rather homogenous group, where moving up or down does not imply large status changes (Fig. 1).

## Conclusion: Status, Wealth and the Middle Class

Wealth inequality, driven by the rising concentration of assets at the very top, has reached new heights in most OECD countries (OECD, 2020). Why do democratic governments not attenuate wealth inequality by increasing wealth redistribution? Although we cannot provide a clear answer to this demanding and puzzling question, this paper sheds light on the public’s preferences on wealth redistribution—an aspect, which has received too little scholarly attention in this context. It was the goal of this paper to better understand how economic concerns shape the public’s preferences for wealth redistribution. Building on the literature of preferences for (income) redistribution, our main argument focused on the role of mobility expectations and how diverging mobility expectations affect the public’s preferences for wealth redistribution. We show that diverging mobility expectations among the lower, middle and upper wealth group undermine the traditional class conflict.

While standard political economy accounts suggest that both the lower and the middle class should be highly supportive of wealth redistribution, we developed an explanation based on mobility expectations for how future prospects can undermine the traditional class struggle over wealth redistribution. In line with the literature, we argued that a person who expects significant wealth gains in the future is likely to be less supportive than an individual with a poor future outlook. Although both hold the same wealth at current, their expectations about which social group they (will) belong to diverge. However, we also suggested that the mobility effect depends on the current wealth class. Due to mostly positive future expectation, the lower class is less likely to push for wealth redistribution, while high financial risks at the top, make the rich more likely to support wealth redistribution. Other than the rich and the poor, the middle class is less responsive to future mobility expectations as they do not feel that changes in the wealth position decisively alter their wealth and status within society. This certainly does not imply that they do not support wealth redistribution, but it does suggest that their economic hopes or fears play no role in their formation of policy preferences.

Based on new public opinion data for fourteen advanced democracies, we explored the public’s preferences toward wealth redistribution in 2019. Although we find that preferences for wealth redistribution generally increase with lower levels in the current and future wealth position, our findings also indicate that public preferences do not perfectly match the traditional class struggle. A key reason for this is that mobility expectations are not evenly distributed among society: In the lower class, future prospects are predominantly positive, in the upper class, they are predominantly negative, and in the middle class, they are two-dimensional. Here, we find similar amounts of individuals who expect to move up- or downward in the wealth distribution. This is a critical finding, as the unresponsiveness of the middle class is not due to no mobility expectation, but instead their preferences do not respond to differences in future prospects.

The findings therefore indicate that the public’s preferences over wealth redistribution are caught in the middle. The future prospects of the middle class have no effects on their preferences for wealth redistribution. People from the middle with poor future prospects have preferences similar to those with positive mobility outlooks. This unresponsiveness is in stark contrast to the effect that mobility expectations have among the lower and the upper classes. Here we find a strong reaction to future mobility expectations. One possible reason for this is that the potential gains and losses for the middle class are somewhat limited as illustrated in Fig. 1. Moving up or down in the middle has little influence on their status and their ability to change their lifestyles. The political consequences of the effect of mobility expectations on preferences for wealth redistribution can, however, be quite far-reaching as they moderate the class conflict over wealth redistribution.

## Appendix

**Fig. 5:**
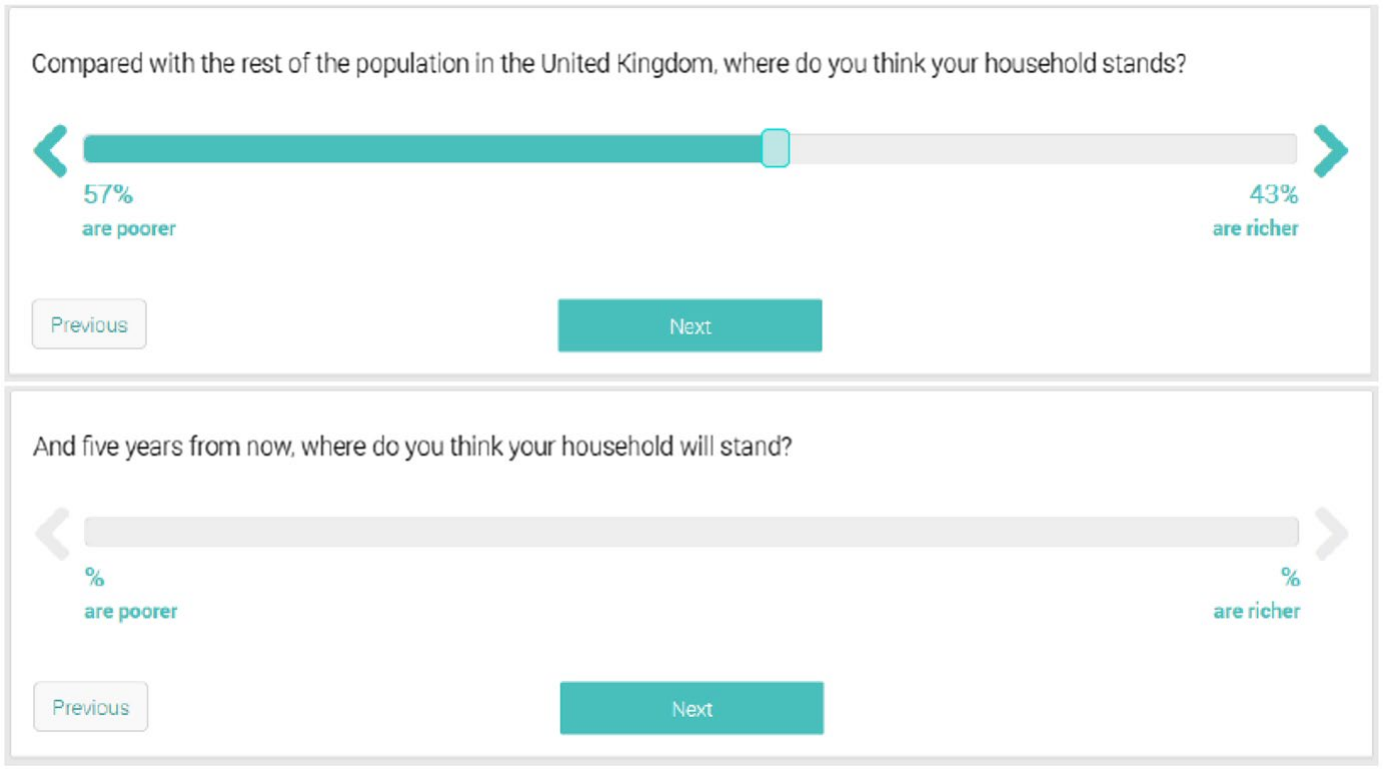
Question on perceived current position and prospect for mobility

See Fig. 5 Tables 2, 3, 4, 5 and 6.

**Table 2:** Linear models with country fixed effects for preferences for wealth redistribution

Mixed effect linear regressions		
	Dependent variable:	
	Preferences for wealth redistribution	
	M1	M2
Perceived position	– 0.015***	
(percentile)	(– 0.014, – 0.010)	
Lower class (ref: middle)		0.595***
		(0.478, 0.669)
Higher class (ref: middle)		– 0.096
		(– 0.228, 0.036)
Prospect of mobility	– 0.010***	– 0.002
(percentile gain)	(– 0.012, – 0.007)	(– 0.006, 0.001)
Lower position		– 0.011***
*Mobility		(– 0.015, – 0.004)
Upper position		– 0.013***
*Mobility		(– 0.020, – 0.006)
Household income decile	– 0.121***	(– 0.135, – 0.107)
	– 0.131***	(– 0.145, – 0.117)
Controls: Gender, age, education, union membership, origin, employment status	✓	✓
Constant	7.499***	6.705***
	(7.261, 7.737)	(6.470, 6.939)
Observations	22,862	22,862
Countries	14	14

*p < 0.05; **p < 0.01; ***p < *0.001*

**Table 3:** Occupation by wealth class

	Lower	Middle	Upper	Total
Prof & technical	11.04%	16.95%	27.67%	16.47%
Higher administrator	2.67%	7.07%	18.62%	7.01%
Clerical	23.40%	28.18%	17.51%	26.19%
Sales	12.81%	11.78%	10.74%	11.93%
Services	15.56%	13.29%	8.84%	13.44%
Skilled worker	10.90%	10.18%	7.41%	10.12%
Semiskilled	7.02%	4.34%	2.33%	4.79%
Unskilled	11.72%	5.47%	4.29%	6.82%
Farm	1.14%	0.76%	1.06%	0.87%
Never worked	3.75%	1.99%	1.53%	2.36%

Source: IAP 2019

**Table 4:** Full model of the linear models with country fixed effects

Mixed effect linear regressions		
	Dependent variable:	
	Preferences for wealth redistribution	
	M1	M2
Perceived position (percentile)	– 0.015***	
	(– 0.017, – 0.013)	
Lower (reference middle)		0.595***
		(0.504, 0.686)
Upper (reference middle)		– 0.096
		(– 0.228, 0.036)
Prospect of mobility (percentile)	– 0.010***	– 0.002
	(– 0.013, – 0.008)	(– 0.006, 0.001)
Lower position*Prospect of mobility		– 0.009***
		(– 0.015, – 0.004)
Upper position*Prospect of mobility		– 0.013***
		(– 0.020, – 0.005)
Household income decile	– 0.121***	– 0.131***
	(– 0.135, – 0.107)	(– 0.145, – 0.117)
Gender (woman)	– 0.114***	– 0.096***
	(– 0.182, – 0.046)	(– 0.164, – 0.027)
Education (university)	– 0.069	– 0.103**
	(– 0.145, 0.006)	(– 0.179, – 0.028)
Age	– 0.004**	– 0.003*
	(– 0.007, – 0.001)	(– 0.006, – 0.0002)
Union membership	0.493***	0.490***
	(0.419, 0.567)	(0.416, 0.564)
Foreign born	0.005	0.004
	(– 0.106, 0.117)	(– 0.108, 0.116)
Employed part– time	0.108*	0.105
	(0.001, 0.215)	(– 0.002, 0.213)
Self-employed	– 0.179*	– 0.202**
	(– 0.323, – 0.035)	(– 0.346, – 0.058)
Unemployed	0.287***	0.283***
	(0.153, 0.420)	(0.149, 0.417)
Full-time parent/homemaker	0.219**	0.215**
	(0.055, 0.382)	(0.052, 0.378)
Full-time student	– 0.150	– 0.150
	(– 0.300, 0.001)	(– 0.301, 0.001)
Retired	– 0.109	– 0.117
	(– 0.228, 0.010)	(– 0.236, 0.002)
Constant	7.499***	6.705***
	(7.261, 7.737)	(6.470, 6.939)
Observations	22,862	22,862
Countries	14	14

*p < 0.05; **p < 0.01; ***p < *0.001*

**Table 5:** Robustness test for model 2 with larger lower and upper wealth classes

Mixed effect linear regressions	
	Dependent variable:
	Preferences for wealth redistribution
Lower position (reference middle)	0.469***
	(0.389, 0.550)
Upper position (reference middle)	– 0.178***
	(– 0.273, – 0.084)
Prospect of mobility (in percentile)	– 0.003
	(– 0.008, 0.001)
Lower position*Prospect of mobility	– 0.006*
	(– 0.012, – 0.001)
Upper position*Prospect of mobility	– 0.010**
	(– 0.017, – 0.003)
Household income decile	– 0.127***
	(– 0.140, – 0.113)
Gender (woman)	– 0.107**
	(– 0.176, – 0.039)
Education (university)	– 0.078*
	(– 0.154, – 0.002)
Age	– 0.003*
	(– 0.006, – 0.0004)
Union membership	0.488***
	(0.414, 0.562)
Foreign born	– 0.0005
	(– 0.112, 0.111)
Employed part– time	0.113*
	(0.006, 0.220)
Self-employed	– 0.185*
	(– 0.329, – 0.041)
Unemployed	0.315***
	(0.182, 0.449)
Full-time parent/homemaker	0.220**
	(0.057, 0.383)
Full-time student	– 0.142
	(– 0.293, 0.009)
Retired	– 0.117
	(– 0.236, 0.001)
Constant	6.664***
	(6.431, 6.898)
Observations	22,862

*p < 0.05; **p < 0.01; ***p < 0.001

Robustness check increases the size of the lower and upper classes by one decile and decreases the size of the middle wealth class by two deciles. The lower-class respondents perceive to be below the 40th percentile (39.5% of the respondents), middle-class respondents between the 41st and the 60th percentile (41.4% of the respondents) and upper-class respondents in the top 39 percentiles (19.1%)

**Table 6:** Robustness test for model 3 with smaller lower and upper wealth classes

Mixed effect linear regressions	
	Dependent variable:
	Preferences for wealth redistribution
Lower position (reference middle)	0.697***
	(0.577, 0.817)
Upper position (reference middle)	0.069
	(– 0.147, 0.284)
Prospect of mobility (in percentile)	– 0.004*
	(– 0.006, – 0.001)
Lower position*Prospect of mobility	– 0.011***
	(– 0.016, – 0.005)
Upper position*Prospect of mobility	– 0.010*
	(– 0.019, – 0.001)
Household income decile	– 0.140***
	(– 0.153, – 0.126)
Gender (woman)	– 0.084*
	(– 0.152, – 0.015)
Education (university)	– 0.116**
	(– 0.192, – 0.041)
Age	– 0.003
	(– 0.006, 0.00004)
Union membership	0.489***
	(0.415, 0.563)
Foreign born	0.009
	(– 0.103, 0.121)
Employed part– time	0.106
	(– 0.001, 0.214)
Self-employed	– 0.209**
	(– 0.353, – 0.064)
Unemployed	0.262***
	(0.128, 0.397)
Full-time parent/homemaker	0.207*
	(0.044, 0.371)
Full-time student	– 0.162*
	(– 0.313, – 0.011)
Retired	– 0.113
	(– 0.232, 0.006)
Constant	6.788***
	(6.554, 7.022)
Observations	22,862

*p < 0.05; **p < 0.01; ***p < 0.001

Robustness check decreases the size of the lower and upper classes by one decile and increases the size of the middle wealth class by two deciles. The lower-class respondents perceive to be below the 20th percentile (12.5% of the respondents), middle-class respondents between the 21st and the 80th percentile (84.4% of the respondents) and upper-class respondents in the top 19 percentiles (3.0% of the respondents)

Based on the IAP Survey Question: Which of the descriptions below best describes the sort of work you do or you used to do when you were working?

1. Professional and technical occupations such as doctor, teacher, engineer, artist, accountant;
2. Higher administrator occupations such as banker, executive in big business, high government official, union official;
3. Clerical occupations such as secretary, clerk, office manager, book keeper;
4. Sales occupations such as sales manager, shop owner, shop assistant, insurance agent;
5. Service occupations such as restaurant owner, police officer, waiter, caretaker, barber, armed forces;
6. Skilled worker such as foreman, motor mechanic, printer, tool and die maker, electrician;
7. Semiskilled worker such as bricklayer, bus driver, cannery worker, carpenter, sheet–metal worker, baker;
8. Unskilled worker such as laborer, porter, unskilled factory worker;
9. Farm worker such as farmer, farm laborer, tractor driver, fisherman;
10. I have never worked.

## Footnotes

1 We categorize those who perceive to fall in lowest 30 percent as belonging to the lower wealth class, those ranking between the 31st and the 70th percent as the middle and those in the top 29 percent as the upper wealth class. To make the middle, the largest group is to account for the fact that this group also tends to be the largest one in advanced democratic societies (OECD, 2019). Other than most sociological approaches, our class definition is not based on different aspects of social status such as education or occupation, but it merely serves the purposes of distinguishing wealth groups. With this, we follow similar accounts on income redistribution (Dallinger, 2013; Lupu and Pontusson, 2011) and apply their approach to the distribution of wealth (see also Sect. 4.1).

2 One reason, which we do not discuss here, is of course that people care about other things than their own material economic position. The role of fairness norms and values is certainly critical, too, but not dealt with in this paper.

3 Please remember, that if not mentioned otherwise, we refer to the *perceived* wealth position.

4 Looking at attitudes, the online respondents from the IAP politics dataset do not seem more liberal than respondents in other well-established surveys. In a replicated question from the ESS2016 and 2018, respondents of the IAP expressed strikingly similar levels of support for redistribution than those in the ESS (Pontusson et al., 2020; ESS, 2016; ESS, 2018).

5 The perceived position varies between 0 and 100, but respondents who answered 0 were recoded into 1.

6 An alternative measure defining the middle class as the three middle quintiles would have shrunk the upper class to 3% of the respondents and the lower to 12%.

7 Results hold with different sizes of wealth classes (see appendix 4).
